# Defining epidemiologically relevant units of cholera transmission in sub-Saharan Africa

**DOI:** 10.1126/sciadv.aeb4683

**Published:** 2026-11-04

**Authors:** Bethany L. DiPrete, Javier Perez-Saez, Shirlee Wohl, Nathaniel Matteson, Seungwon Kim, Andrew S. Azman, Justin Lessler

**Affiliations:** ^1^ Department of Epidemiology, Gillings School of Global Public Health, University of North Carolina at Chapel Hill, Chapel Hill, NC, USA.; ^2^ Department of Epidemiology, Bloomberg School of Public Health, Johns Hopkins University, Baltimore, MD, USA.; ^3^ Geneva Centre for Emerging Viruses and Division of Tropical and Humanitarian Medicine, Geneva University Hospitals, Geneva, Switzerland.; ^4^ Brigham and Women’s Hospital, Harvard Medical School, Boston, MA, USA.; ^5^ Carolina Population Center, University of North Carolina at Chapel Hill, Chapel Hill, NC, USA.

## Abstract

Much remains unknown about cholera transmission dynamics in Africa. Applying both traditional phylogeographic techniques and innovative methods for combining epidemiologic and genomic data, we identified three to four distinct “cholera transmission units” within which cross-border cholera transmission has been more frequent. These units can be grouped into two major transmission clusters, one in Western Africa and bordering countries and one spanning the Central, Eastern, and Southern regions. Simulation studies show that these clusters can be used to assess the risk of international spread of introduced cholera strains and identify heterogeneities in the speed of international spread based on outbreak location. Our methods further allow us to infer cholera incidence attributable to specific lineages in the absence of sequencing data. This work has clear implications for cholera control.

## INTRODUCTION

Cholera is an acute gastrointestinal infection caused by toxigenic *Vibrio cholerae* bacillus. Disease is characterized by watery diarrhea and rapid dehydration, which can rapidly progress to death if untreated. Despite a multitude of proven tools for cholera control, including improved water and sanitation infrastructure, vaccines, and effective treatment with oral rehydration salts, cholera continues to cause substantial morbidity and mortality, with an estimated 1.3 to 4.0 million cases and 21,000 to 143,000 deaths annually (*1*). The disease is widespread in its ancestral home in countries surrounding the Bay of Bengal (*2*–*4*); however, much of the world’s cholera mortality is concentrated in sub-Saharan Africa (*5*).

Cholera has been endemic in sub-Saharan Africa since the 1960s when pandemic O1 El Tor *V. cholerae* was introduced from Southeast Asia (*2*). Incidence in the region tends to follow seasonal patterns (*6*, *7*), but large, often off-season outbreaks are not uncommon and can have devastating impacts (*8*, *9*). Natural disasters and social disruptions often trigger major cholera epidemics (*10*, *11*) but do not explain cholera’s persistence and spread throughout the continent. To uncover the underlying drivers of cholera spread, scientists have turned to whole-genome sequencing of *V. cholerae* and found that there have been at least 17 introductions of O1 El Tor *V. cholerae* (lineages AFR1 to AFR17) from Southeast Asia into Africa since 1970 (*12*, *13*). Recent studies provide some insight into how cholera moves throughout the continent once introduced, finding evidence for frequent cross-border transmission between nearby countries, strain cocirculation within countries, and long-term persistence of lineages during epidemic lulls (*13*–*15*). However, these studies have been limited in their geographic and temporal scope, in part due to the limited and uneven coverage of available sequence data (Fig. 1A). Hence, questions remain about the geographic structure of cholera transmission within the continent, lineage persistence, and how newly introduced lineages are likely to spread.

**Fig. 1.**
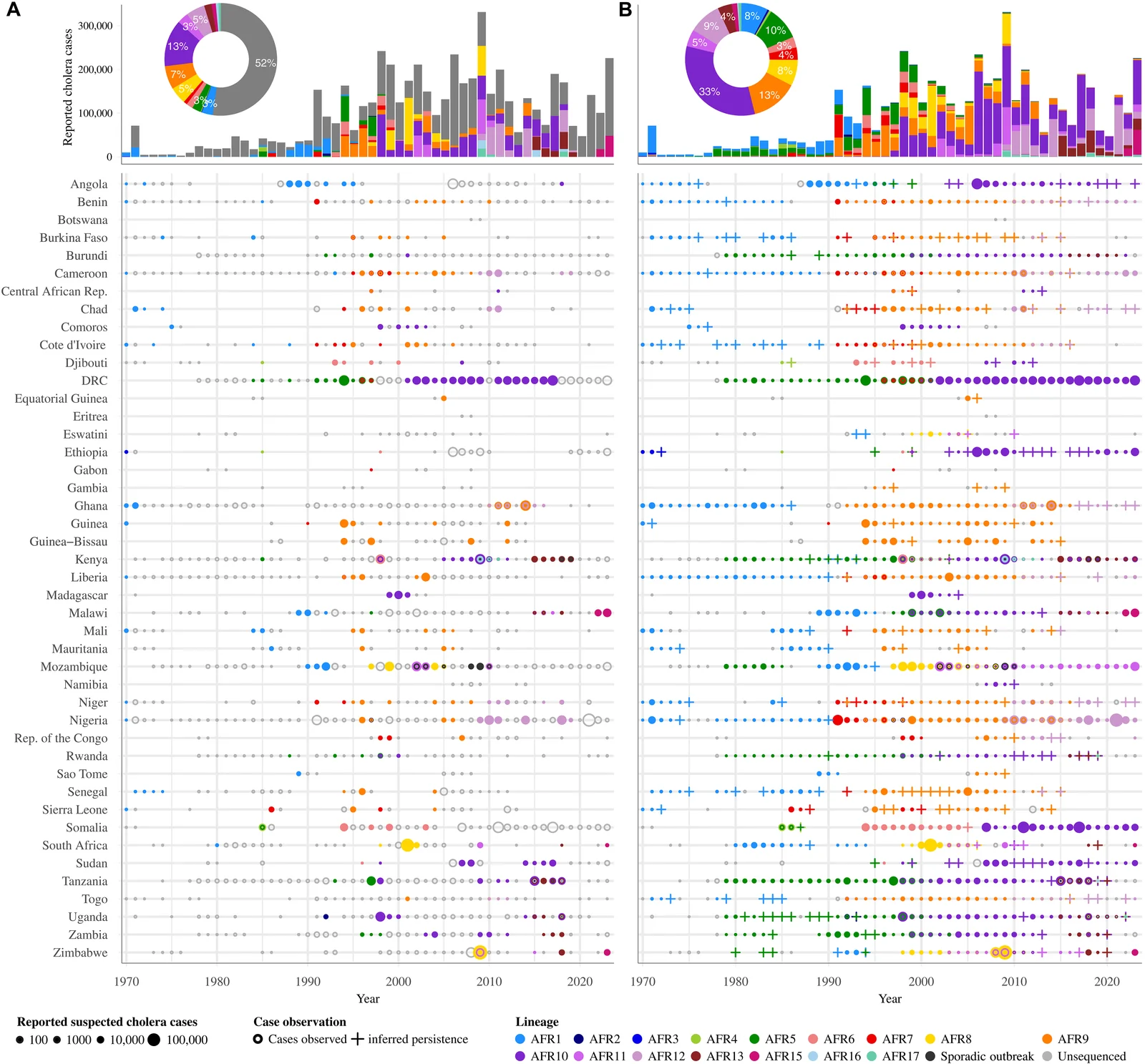
Reported cholera cases and lineages by country, 1970–2023. (**A**) Reported suspected cholera cases, depicted by the size of the circle, and detected lineages, indicated by the color of the circle. Multiple colors within a circle indicate >1 lineages detected through sequencing, whereas an empty gray circle indicates no sequences available. Bar graph displays total cases by year attributable to each lineage, and donut plot shows the proportion of cases attributable to each lineage overall. (**B**) Reported suspected cholera cases, depicted by the size of the circle. Detected lineages and lineages inferred through modeling are indicated by the color of the circle, where multiple colors within a circle indicate >1 lineages inferred or detected through sequencing. Crosses indicate inferred lineage presence when no cases were reported. Bar graph displays total cases by year attributable to a given observed or inferred lineage, and donut plot shows the proportion of cases attributable to each lineage overall.

## RESULTS

### Phylogeographic analysis

We used phylogeographic techniques to infer international cholera transition rates within Africa based on 1762 third-wave (*16*) pandemic (AFR9 onward) cholera sequences (table S1) collected from 36 African countries between 1989 and 2023 (Figs. 2 and 3, A and B; see Materials and Methods). We observed the highest density and magnitude of geographic transitions between spatially proximal countries, with infrequent support for transitions between more distant locations (Fig. 3A), consistent with previous regional studies (*13*–*15*, *17*). For instance, with every additional 1000-km distance between country centroids, the probability of a supported transition decreased by 88% [OR 0.12; 95% confidence interval (CI) 0.07, 0.17].

**Fig. 2.**
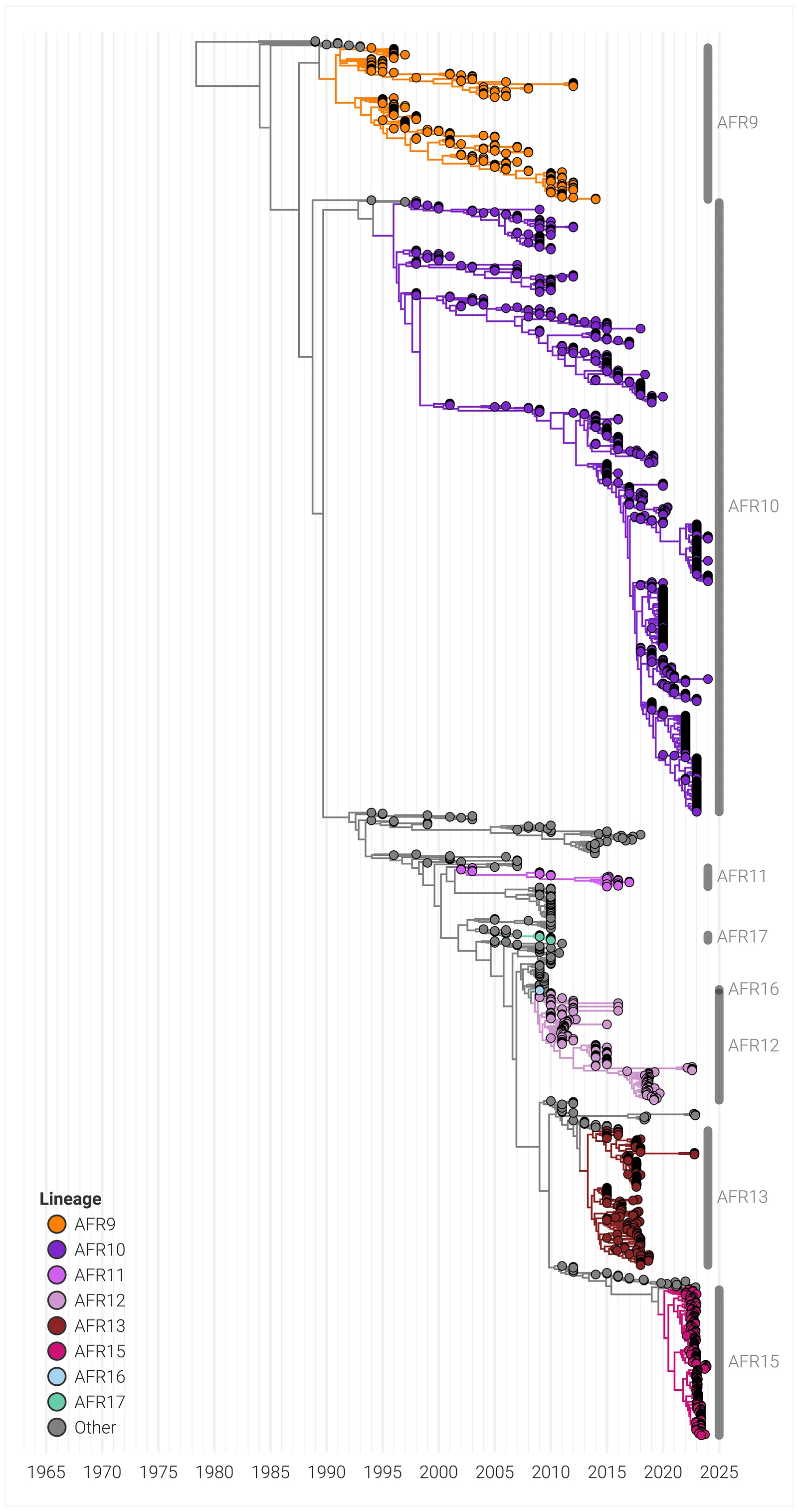
Phylogeny of third-wave seventh pandemic *V. cholerae* El Tor genomes. Maximum likelihood phylogeny of 1762 third-wave genomes from 1989 to 2023 included in the phylogeographic analysis, with tip colored to indicate the inferred lineage.

**Fig. 3.**
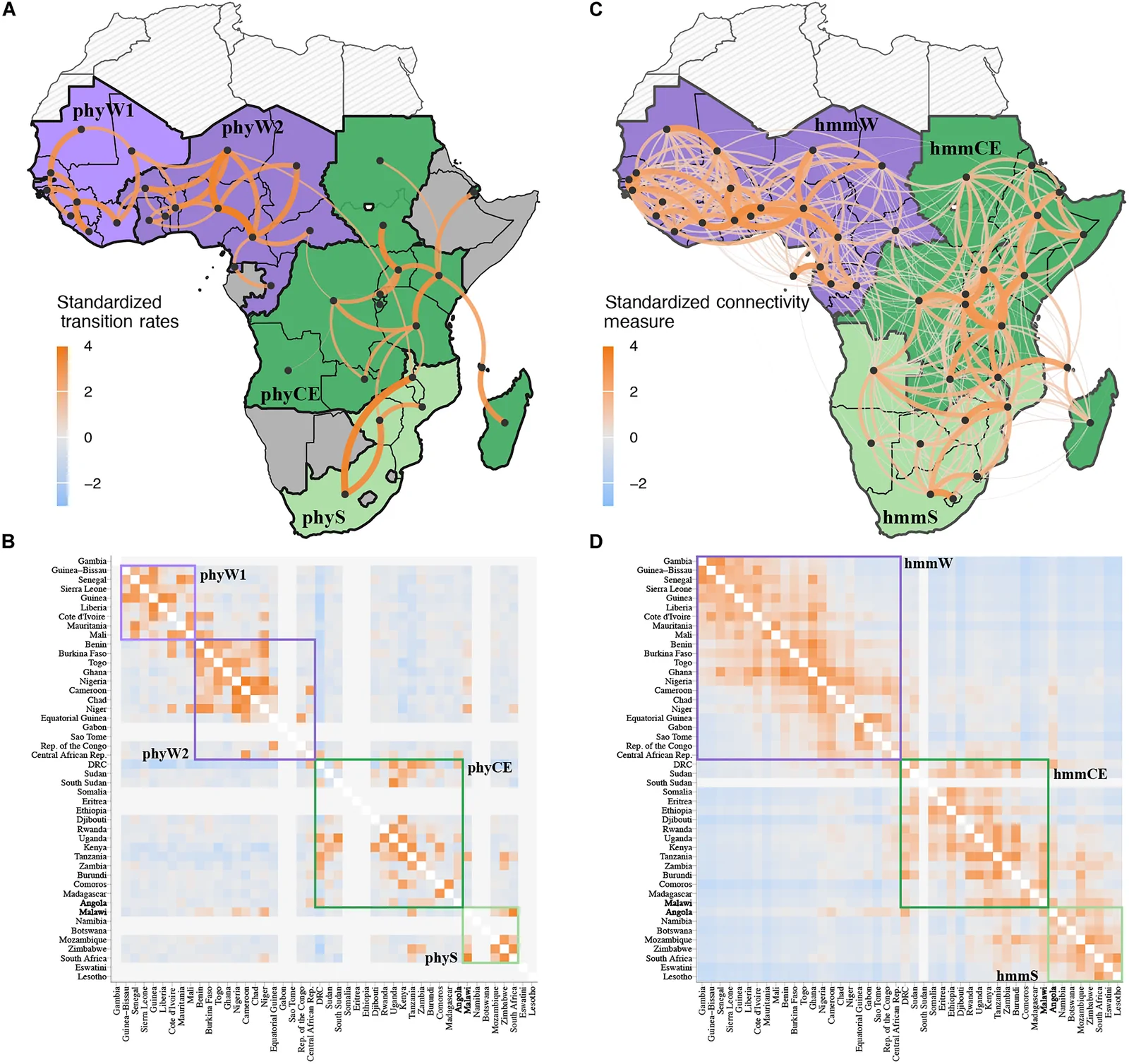
Inferred cholera transmission units and cross-country transmission in sub-Saharan Africa. (**A**) Results from phylogeographic analysis. Four identified cholera transmission units are shown by fill color (dark purple, phyW1; lighter purple, phyW2; dark green, phyCE; light green, phyS), with the color and thickness of edges representing the standardized transition rates between countries. Only edges with model support in 50% of runs are shown. Hashed gray indicates countries in Northern Africa that are not included in any analyses, whereas countries with the darker gray fill are not included in the phylogeographic analysis. (**B**) Heatmap of standardized transition rates between countries using the full network. Bolded countries are in a different order than (D) due to clustering. (**C**) Results from the HMM model. Three identified cholera transmission units are shown by fill color (purple, hmmW; dark green, hmmCE; light green, hmmS), with the color and thickness of edges representing the standardized connectivity measure between countries. Only the top 50% of edges are plotted. Hashed gray indicates countries in Northern Africa that are not included in any analyses. (**D**) Heatmap of the standardized connectivity measure between countries using the full network. Bolded countries are in a different order than (B) due to clustering.

We identified four distinct, but interconnected, “cholera transmission units” defined by the probability of intra- versus extra-unit cholera transmission (Fig. 3, A and B). We first extracted nonoverlapping communities from the network of transition rates between countries for each posterior sample using the Louvain method of community detection (*18*). On the basis of how consistently countries clustered together (fig. S1A and Materials and Methods), we then identified four cholera transmission units (*19*), two in Western Africa and neighboring countries (phyW1 and phyW2), one in Central and Eastern Africa (phyCE), and one in Southern Africa (phyS) (Fig. 3, A and B). Transition rates within cholera transmission units were, on average, 13.5 (95% HPD interval: 9.7, 17.6) times that between cholera transmission units. We were able to further group these transmission units into a Western cluster (including phyW1 and phyW2) and one spanning Central, Eastern, and Southern Africa (including phyCE and phyS) based on cross-unit transition rates (rates between phyW1 and phyW2 and between phyCE and phyS were 2.6 [1.3, 3.9] and 2.1 [1.0, 3.4] times the rates between the Western cluster and Central/Eastern/Southern cluster, respectively).

While this phylogeographic analysis provides important insights into cholera transmission patterns, it is limited by its reliance solely on sparse and unevenly distributed available sequence data (*20*–*22*). This analysis focused solely on third-wave data because, before 1989, sequence data were only available for 14% of country-year combinations where cholera was reported to the WHO. Even within the period covered, we have third-wave sequence data for only 22% of country-year combinations, and no cholera sequence data are available for 9 of 45 sub-Saharan African countries (Fig. 1A). Sparse coverage means that we are only able to infer infecting lineages based on local, contemporaneous, sequence data for 48% of African cholera cases since 1970. Hence, if we are to gain deeper insights into cholera transmission on the continent spanning the entirety of the seventh cholera pandemic, we must find a way to leverage the more widely available data on cholera incidence.

### Combining epidemiologic and lineage data in a hidden Markov modeling approach

We developed an innovative modeling approach that leverages epidemiologic data and sequence-derived cholera lineages to infer missing cholera lineages and gain further insights into African transmission dynamics. We used a hidden Markov model (HMM) to synthesize incidence data from 1970 to 2023, covering 45 African countries with available lineage information from local, contemporaneous sequences (26% of country-years with reported cases). We modeled the underlying transmission and observation process of cholera to estimate the probability of lineage presence (its hidden presence state) across space and time and the probability of lineage movement between pairs of countries (cross-border connectivity; see Materials and Methods). Briefly, our modeling approach for the probability of lineage presence incorporated importations from outside of the continent and onward transmission based on outbreak size and lineage presence.

#### Inferring presence, prevalence, and persistence of infecting lineages

Using this approach, we were able to infer infecting lineages for all reported cholera cases in sub-Saharan Africa (Fig. 1B). We estimate that 46% of the cholera cases observed in Africa since 1970 (2.4 million cases) can be attributed to two lineages, AFR10 (33% [95% CI: 31, 35]) and AFR9 (14% [95% CI: 12, 15]), with no other lineage accounting for more than 10% of reported cases (Fig. 1B). This analysis also gives further insight into local dynamics when sequence data are limited, e.g., suggesting the displacement of AFR5 by AFR10 in East Africa in the late 1990s and early 2000s was a trans-national phenomenon and showing AFR10s persistence in these countries during periods with few cases and no sequence data (Fig. 1B and fig. S2).

#### Defining cholera transmission units—HMM model

Using inferred connectivity from the HMM model, we identified three distinct cholera transmission units largely consistent with the phylogeographic analysis. Applying methods similar to those used to identify consensus clusters above to inferred connectivity measures (*18*, *19*), we identified three cholera transmission units (Fig. 3, C and D, and fig. S1B): one encompassing Western Africa and bordering countries (hmmW), a second spanning Central and Eastern Africa (hmmCE), and a third largely in Southern Africa (hmmS). As with the phylogeographic analysis, these cholera transmission units further grouped into two clusters, a Western cluster (hmmW) and one spanning Central, Eastern, and Southern Africa (hmmCE and hmmS). These major clusters were identical to those found in the phylogeographic analysis, while the individual cholera transmission units only differed in there being one (hmmW) versus two (phyW1 and phyW2) Western transmission units and the assignment of Angola to the Southern (hmmS) rather than the Central/Eastern (hmmCE) transmission unit and of Malawi to hmmCE rather than hmmS. Lineage movement between countries was 7.8 times (95% CI 6.5, 9.4) more likely within cholera transmission units (versus between). The consistency of these results across methods affirms the distinctness of the Western cholera epidemic from the rest of the continent and shows the consistency of transmission patterns over time (clusters were similar when data were subset to 1990 onward and 2000 onward, particularly the distinctness of the Western cluster; see fig. S3).

#### Simulating onward spread of cholera

Simulations of the spread of newly introduced lineages made possible by the HMM model further support the importance of cholera transmission units. We used the HMM model to simulate onward spread from 2000 introductions of cholera into each of 45 countries (sampling parameters from the posterior for each, see Materials and Methods). We found that within the first year after a simulated introduction into a single country, countries within the same cholera transmission unit were 10.3 times (95% CI 10.1, 10.5) more likely to experience a subsequent cholera outbreak compared to countries outside of that cholera transmission unit (Fig. 4 and fig. S4). Similarly, the average time to arrival of a newly introduced strain to countries within the same transmission unit was 3.3 years lower (95% CI −3.2, −3.2) than the average time to arrival for countries outside of that unit (Fig. 5 and fig. S5). Transmission between the Central/Eastern cholera transmission unit (hmmCE) and the Southern transmission unit (hmmS) was also more likely than between those units and the Western transmission unit (hmmW). For instance, an introduction in hmmCE is 4.4 times (95% CI 4.2, 4.7) more likely to cause an outbreak in hmmS in the next year versus causing one in hmmW.

**Fig. 4.**
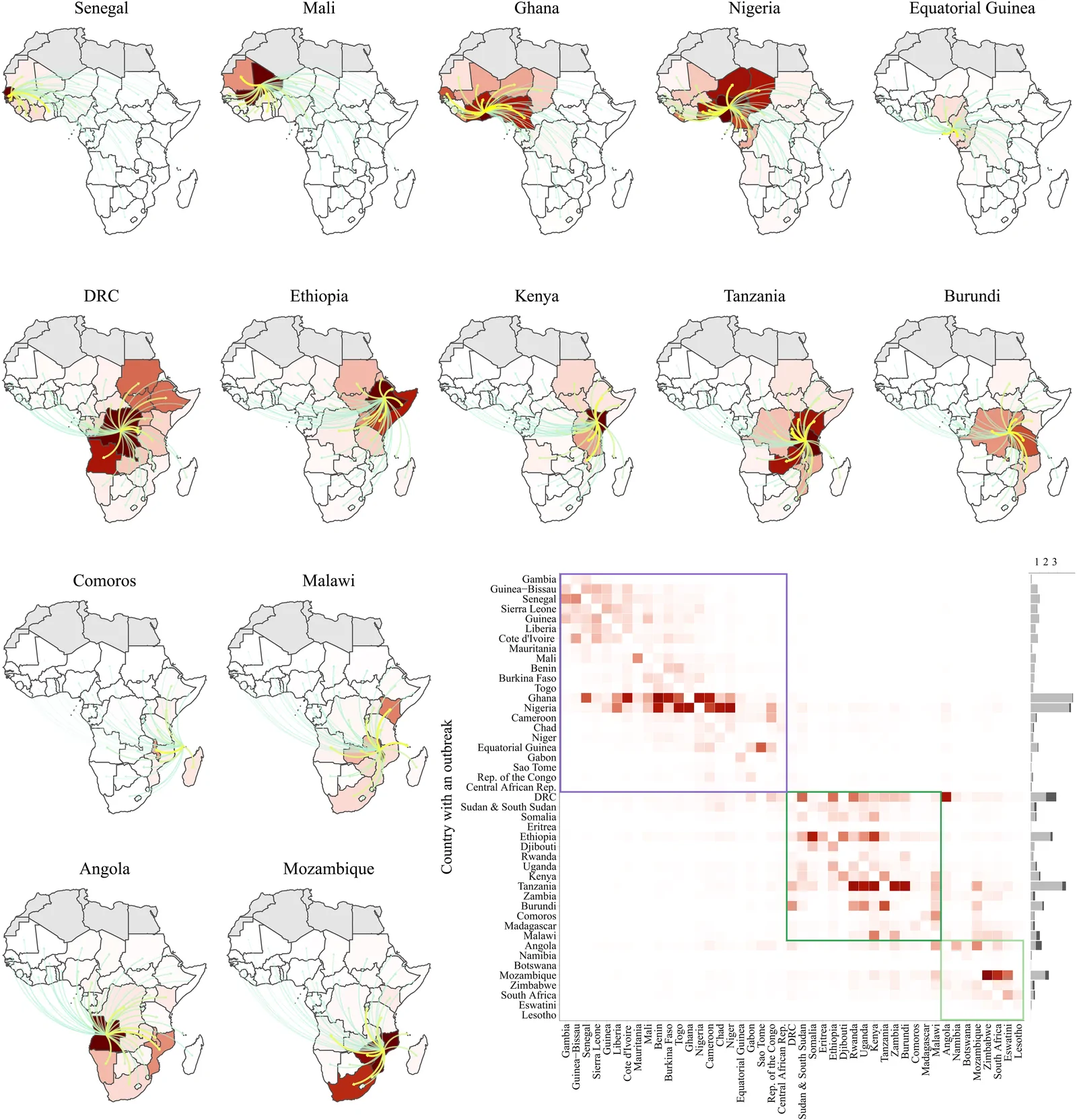
One-year risk of cholera outbreak by country of introduction. Simulated 1-year risk of cholera outbreaks from 2000 introductions of cholera into each of 45 countries (sampling parameters from the posterior for each), with darker red indicating higher risk of an outbreak in the following year. Edges indicate the asymmetrical strength of connectivity between the country of introduction and the rest of the continent. Maps are shown for a subset of countries with the highest potential for seeding outbreaks. The heatmap displays outbreak risk across all countries, with the side panel showing the expected number of additional outbreaks caused within (light gray) and between (dark gray) transmission units by country of introduction.

**Fig. 5.**
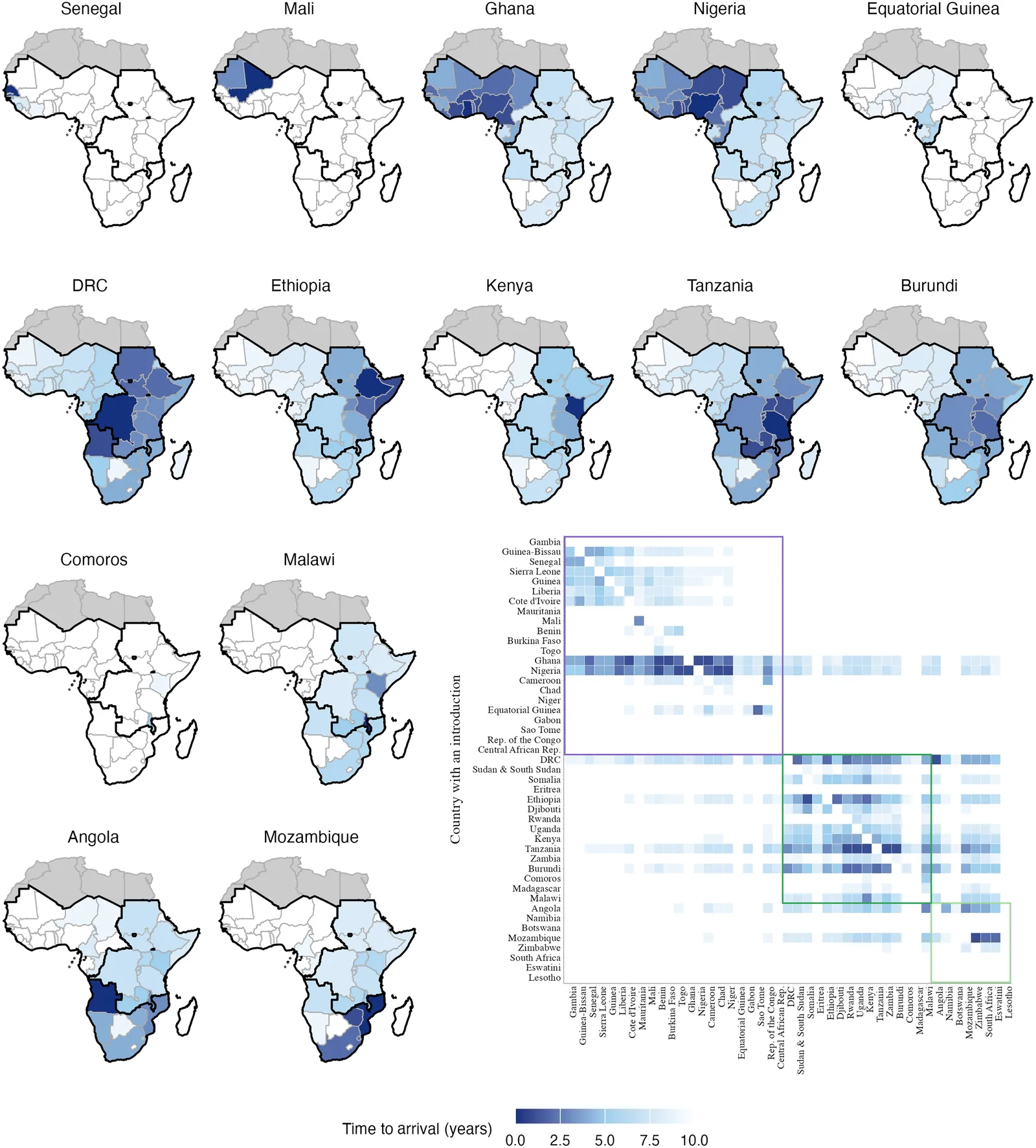
Speed of cholera spread by country of introduction. Simulated downstream time to arrival of cholera from 2000 introductions of cholera into each of 45 countries (sampling parameters from the posterior for each). Maps are shown for a subset of countries with the highest potential for seeding outbreaks, whereas the heatmap shows time to arrival across all countries by country of introduction.

Even within transmission units, there are stark differences in the predicted extent and speed of spread from each country (Figs. 4 and 5 and table S2). For example, an introduction into Ghana would be expected to cause outbreaks in 4.69 other countries within the Western cholera transmission unit (hmmW) within 1 year and be expected to reach at least 50% of the countries within that unit within 4.2 years. Similarly, an introduction into Nigeria would be expected to cause outbreaks in 4.33 countries in hmmW. However, an introduction into neighboring Cameroon would only be expected to cause outbreaks in 0.58 countries in hmmW within a year and would take 10 years to spread across even a quarter of the cholera transmission unit. Likewise, some countries are more prone to spreading cholera between cholera transmission units than others. For instance, an introduction into DRC (in hmmCE) would be expected to cause 1.14 additional outbreaks in a different cholera transmission unit within a year (0.43 in hmmW and 0.71 in hmmS), while neighboring Kenya would only be expected to cause 0.15 such outbreaks (0.07 in hmmW and 0.08 in hmmS).

## DISCUSSION

These results enrich our understanding of cholera transmission within Africa and have clear implications for assessing cholera risk and thinking about country-level cholera control. The existence of cholera transmission units provides a framework for anticipating which countries are likely most at risk from cholera outbreaks elsewhere on the continent and the natural grouping of countries over which we can coordinate cholera control. For instance, Cameroon might benefit more from coordinating cholera control efforts with Ghana (also in hmmW) than it would with the Democratic Republic of the Congo (in hmmCE), despite being geographically closer to the latter. Likewise, in response to a large cholera outbreak in Angola (hmmS), prophylactic vaccination would be better targeted in Mozambique (hmmS) than in the Republic of the Congo (hmmW). Our speed-of-spread simulations, although focused on newly introduced strains, provide some insight into country-to-country variation in the international spread of cholera resulting from outbreaks of all types. They suggest that outbreaks in some countries (e.g., Mozambique, DRC) are more likely to seed outbreaks elsewhere on the continent, while those in others (e.g., Senegal, Comoros) are likely to remain self-contained. This may help to further refine risk assessment for the distribution of prophylactic vaccines from the global stockpile and other interventions.

Our analysis of cholera transmission dynamics is not without limitations. As noted, the phylogeographic analysis was limited by data sparsity. Although the HMM modeling approach allowed us to infer much of these missing data, its inferences were also limited by data availability (as evidenced by improved performance in validation experiments in later years, see below) and came at the expense of losing valuable information on the genetic relationships between outbreaks of the same lineage. Further, inferences were based on reported suspected cholera cases, which are known to be an imperfect measure of true incidence (*1*), and we used time-fixed priors to account for case underreporting by country. While we incorporated intercontinental importations into our model, we relied on inferred timing and locations of lineage introductions, and our understanding of importation events is inherently limited by sparse sampling (*12*, *13*). The use of country-level data and centroid-based distances likely obscures critical aspects of cholera dynamics. This may be particularly true in the DRC, where cholera epidemics in the east and west of the country are known to be relatively independent (*23*), and most cases occur in the eastern region of DRC, which is likely more connected to Eastern Africa. Using subnational instead of country-level data could reveal further nuances in inter- and intracountry transmission dynamics. Further, because of complexities in disentangling and modeling cases and lineages in Sudan and South Sudan before and after 2014, we combined them geographically for HMM analyses while recognizing that the two countries are fully politically separate and may have different transmission dynamics. Our analysis takes no explicit account of immunity or strain-strain competition, which, along with other modeling assumptions, may affect the interpretation of our findings regarding lineage persistence, the spread of lineages, and lineage replacement. Last, our HMM analysis covered an extended time period during which cholera transmission dynamics, surveillance intensity, and case reporting practices may have changed on the continent. This appears to only have a marginal effect as the results are largely consistent when we subset data to 1990 or 2000 onward, particularly regarding the boundary between hmmW and hmmCE (figs. S3 and S6) but may explain some of the differences between the phylogeographic and HMM analyses, as well as small shifts in the boundaries between hmmCE and hmmS over time.

Despite these limitations, validation experiments show the robustness of our modeling approach. In cross-validation experiments where we removed 20% of detected lineages across countries and years (see Materials and Methods), the HMM model could infer detected lineages with 73% (SD = 7) recall (table S3). Later, more densely sampled lineages were generally recovered more consistently (e.g., 94 and 87% recall for AFR10 and AFR13, respectively). If we presume clonal outbreaks (unless cocirculation is observed), precision was 65% (SD = 7); however, undetected cocirculation cannot be ruled out (and is assumed to be possible throughout the analysis); hence, this may overestimate error (precision assuming nonclonal outbreaks: 85%, SD = 8). In simulation experiments where we compared the simulated time until a lineage would reach a country based on the country of introduction with observed arrival times (fig. S7; see Materials and Methods), we found moderate agreement between predicted and observed arrival times, both when the latter was based on the first observation of a lineage (Pearson *r* = 0.49) and when based on the inferred arrival times from the full model fit (*r* = 0.55). Predictions were largely more accurate for later or more densely sampled lineages (e.g., *r* = 0.91 for observed AFR13). An advantage of this analysis is that it is based on actual cholera transmission patterns rather than being inferred from external sources.

This work illustrates the power of combining molecular and epidemiologic data to gain consequential insights into disease ecology (*17*). Phylogeographic analyses based on full-genome sequences can provide a detailed understanding of the complex relationships between pathogen lineages, thereby revealing the ecologic and epidemiologic processes that create them. However, the sequences underlying these analyses will almost always be a biased and incomplete sample of the underlying pathogen population; hence may not give us a complete or fully accurate picture. By pairing phylogeographic analysis with complementary inferential methods that leverage both (less detailed) molecular relationships and epidemiologic data, we were able to gain deeper insights into cholera transmission in Africa. Our observations of clearly defined cholera transmission units, as well as inferences about lineage persistence and onward spread, can both inform public health action and drive further scientific enquiry. We also hope that this work can serve as a template for tighter integration of molecular and epidemiologic data in the study of other pathogens.

## MATERIALS AND METHODS

### Data sources

Sequence data were compiled from published sequences from GenBank and VibrioWatch from 1970 to 2023 (table S1). This curated list of cholera sequence data also contained metadata on country, year, and lineage call. Cholera case data for this analysis came from the WHO’s database of cholera cases reported by Member States, which contains annual reported suspected and confirmed cholera cases by country from 1970 to 2023.

### Phylogeographic analysis

We used the phylogeny subcommand of bacpage (Bacterial Genomic Analysis Pipeline; github.com/CholGen/bacpage) to generate a recombination-masked maximum likelihood phylogeny. Briefly, the pipeline concatenated individual reference-based assemblies along with the N16961 reference into a pseudo-alignment. Problematic sites including known recombinant regions, repetitive sequences, and homoplastic sites were masked on the basis of the GFF file provided by Weill *et al.* (*12*). Recombinant regions and regions with significantly elevated substitution densities were masked using Gubbins v2.3.4 (*24*). To reduce the computational burden of phylogenetic analysis, we filtered the pseudo-alignment to include only variable positions using SNP sites v2.5.1 (*25*). We then constructed a maximum likelihood phylogenetic tree using IQ-TREE2 (*26*) with a GTR substitution model (*27*).

The resulting phylogeny was rooted on the reference genome. To further reduce computational burden, we extracted the subtree descending from the most recent common ancestor of lineages AFR9 to AFR15, corresponding to the third wave of 7PET. This subtree was time-resolved using Treetime v0.11.3 (*28*). We pruned taxa that deviated more than three interquartile ranges from the clock-rate regression from the phylogeny. In addition, we randomly resolved polytomies by adding zero-length branches with gotree v0.4.5 (*29*).

We refined the time-resolved phylogeny using BEAST X v10.5.0 (beta 5) (*30*). We specified a GTR substitution model with gamma-distributed rate heterogeneity under an uncorrelated relaxed molecular clock and a constant coalescent tree prior. For the uncorrelated relaxed molecular clock, we specified an informative prior consisting of a log-normal distribution with a mean of 8.4 × 10^−7^ substitutions/site per year and an SD of 1.4 × 10^−6^, consistent with parameters reported by Weill *et al.* (*12*).

We ran a Markov chain Monte Carlo (MCMC) chain of 300 million steps using the BEAGLE computational library (*31*), sampling parameters and trees every 10,000 and 100,000 states, respectively. We assessed the convergence and mixing of the MCMC chain with Tracer v1.7.2 (*32*) and confirmed that all estimated continuous parameters achieve effective sample sizes of greater than 100.

We performed a discrete state ancestral reconstruction on geographic states using BEAST. This analysis reconstructed location-transition history across an empirical distribution of 2000 time-calibrated trees sampled from the posterior tree distribution estimated above (*33*, *34*). For this analysis, we assigned each taxon a discrete location state based on its country of collection. To limit the number of transition rates requiring estimation, we binned non-African sequences into a single “Other” state. We assumed that geographic transition rates were reversible and used a symmetric substitution model. We used Bayesian stochastic search variable selection to infer nonzero migration rates (*35*). The MCMC algorithm ran for 500,000 generations, and transition rates were sampled every 500 generations. We used TreeAnnotator v1.10 to construct a maximum clade credibility tree, which we visualized using Baltic (https://github.com/evogytis/baltic).

### Modeling presence and prevalence of cholera lineages (HMM model)

We modeled the probability of the presence of distinct cholera lineages in countries in sub-Saharan Africa through time using an HMM. Our targets of interest were (i) the strength of connectivity driving cholera transmission between locations and (ii) the underlying presence and prevalence of cholera lineages in each country across time. We considered discrete, hidden states, $z_{v,i,t}\in \left\{0,1\right\}$, representing the absence or presence of a cholera lineage (v) in a given country (i) and year (t), focusing on the probability of lineage presence, $\text{Pr}\left(z_{v,i,t}=1\right)$ (see table S4 for the list of parameters and interpretations).

For modeling purposes, we made the following assumptions: (i) no competition between lineages, (ii) introduction of a lineage into a given country is independent of other lineages already circulating in that country, (iii) the probability of a lineage being introduced or reintroduced from another country is a function of the number of reported cholera cases in that country and on the distance separating the two countries, (iv) perfect specificity of sequencing $\left[\text{Pr}\left(y_{v,i,t}\geq 1|z_{v,i,t}=0\right)=0\right]$, and (v) introduction of lineage v to sub-Saharan Africa is based on inferred introduction timing and initial locations of circulation. In addition, because of complexities in disentangling and modeling cases/lineages in Sudan and South Sudan before and after 2014, we geographically combined them in analyses, fully recognizing that the two countries are politically separate and may have different transmission dynamics.

We constructed our model, H={π,Φ,Ψ}, as follows

$$\pi_{v,i,1}:\text{initial probability of presence},\text{Pr}\left(z_{v,i,1}=1\right)$$

$$\Phi :\text{transition process},\Phi_{k,l}=\text{Pr}(z_{v,i,t}=1|z_{v,1,t-1},\ldots ,z_{v,M,t-1})$$

$$\Psi :\text{observation process},\psi_{l}=\text{Pr}(y_{v,i,t}|z_{v,i,t})$$

#### Initial probability of presence

The initial probability of presence, $\pi_{v,i,1}$, was inferred on the basis of lineage observation, using priors assuming (i) high probability of presence if the lineage was observed in the country of interest, (ii) moderate probability if observed elsewhere, (iii) moderately low probability if inferred to have been introduced to the continent before 1970, and (iv) low probability if not observed on the continent and not inferred to have already been introduced to the continent (see the “Parameter priors” section below).

#### Observation process

The observed data are the number of sequenced samples of lineage *v* in country *i* and year *t*, $y_{v,i,t}$, and reported suspected cholera cases in country *i* and year *t,* $c_{i,t}$.

*Sequence data.* The observation process for sequence data, Ψ, is $Y_{v,i}=\left[Y_{v,i,t},t=1,\ldots ,T\right]$, is the observation of the number of sequenced samples of lineage *v* in country *i* and year *t*, and is associated with the hidden process, $\Phi =\left(\Phi_{v,i,t}^{k,l},t=1,\ldots ,T\right)$, of the underlying true presence of lineage *v* in country *i* and year *t* (see the “Transition process” section below). To estimate lineage prevalence, we modeled the probability of our observed sequence data ($y_{t}$) given the unobserved (hidden) states of presence ($z_{v,i,t}=1$) or absence ($z_{v,i,t}=0$) of lineage *v* in country *i* at time *t*

$$\text{Pr}\left(y_{v,i,t}|z_{v,i,t}\right)=\left\{\begin{array}{cc} \left(1-\rho_{v,i,t}\right)+\rho_{v,i,t}\text{Pr}\left(y_{v,i,t}=0|\lambda_{v,i,t}\right) & \text{if}\;y_{v,i,t}=0\\ \rho_{v,i,t}\text{Pr}\left(y_{v,i,t}|\lambda_{v,i,t}\right) & \text{if}\;y_{v,i,t}>0 \end{array}\right.$$ (1)

where $\rho_{v,i,t}=\text{Pr}\left(z_{v,i,t}=1\right)$ and $\lambda_{v,i,t}$ is the number of sequences attributable to lineage *v* in country *i* and year *t*. We use the Poisson approximation of the multinomial (*36*) in our observation process for sequenced cholera lineages

$$Y_{v,i,t}∼\text{Poisson}\left(\lambda_{v,i,t}\right)$$

$$\text{log}\left(\lambda_{v,i,t}\right)∼\text{Normal}\;\left(\text{log}\left[\frac{I\left(c_{i,t}>0\right)\bar{y_{i,t}}\;E\left(z_{v,i,t}\right)}{E\left(\sum_{q\neq v}z_{q,i,t}|z_{v,i,t}=1\right)}\right],\sigma \right)$$ (2)

$$\lambda_{v,i,t}^{*}=\frac{\lambda_{v,i,t}}{\sum_{v}\lambda_{v,i,t}}$$

*Reported suspected cholera cases.* We also modeled the observation process for cholera cases ($c_{i,t})$

$$\text{Pr}\left(c_{i,t}|z_{v,i,t}\right)=\left\{ \begin{array}{cc} \left(1-\alpha_{i,t}^{+}\right)+\alpha_{i,t}^{+}\text{Pr}\left(c_{i,t}=0\left|\frac{1}{\varepsilon_{i}}+N_{i,t}\right.\right) & \text{if}\;c_{i,t}=0\\ \alpha_{i,t}^{+}\text{Pr}\left(c_{i,t}\left|\frac{c_{i,t}}{\varepsilon_{i}}\right.\right) & \text{if}\;c_{i,t}>0 \end{array}\right.$$ (3)

where $\varepsilon_{i}$ is case underreporting by country, and $N_{i,t}$ is the total number of sequences collected by country and year. Given that there are a small number of instances where we observe sequences in countries and years with no reported cases, we handle these instances by assuming that there are at least as many cases as there are observed sequences, allowing for underreporting. In addition, $\alpha_{i,t}^{+}$ is the probability of any lineage being present, based on the forward algorithm

$$\alpha_{i,t}^{+}=1-\prod_{v}1-\alpha_{v,i,t}$$ (4)

#### Transition process

We modeled the transition process between presence states as a function of within-country persistence, extracontinental introductions, and estimated trans-national connectivity. Specifically, for the transition process, Φ, probability of lineage presence is based on the probability of establishment or persistence of lineage *v* in country *i* at time *t*. The probability of presence in time t given the state in time t−1 is based on the transition matrix **A** = $\left\{\Phi_{v,i,t}^{k,l}\right\}$, where $\Phi_{v,i,t}^{k,l}=\text{Pr}(z_{v,i,t}=1|z_{v,1,t-1},\ldots ,z_{v,M,t-1})$, and is a function of the introduction/reintroduction rate, $\phi_{v,j,t}$ from other countries. Because we focus on the probability of lineage presence, we define$\Phi_{v,i,t}=\text{Pr}(z_{v,i,t}=1|z_{v,i,t-1}=k)$

$$\Phi_{v,i,t}^{k,l}=1-\left[\left(1-\gamma_{v,i,t}\right)\prod_{j}e^{-\phi_{v,j,t}}\right]$$ (5)

where

$$1-e^{-\phi_{v,j,t}}=\text{Pr}\left(z_{v,i,t}=1|z_{v,j,t-1}=k\right)$$ (6)

$$\phi_{v,j,t}={\left(\lambda_{v,j,t-1}^{*}c_{j,t-1}\right)}^{\eta }\xi_{j,i}$$ (7)

where introduction to country *i* is based on introduction from outside of the continent, $\gamma_{v,i,t}$, and introduction/reintroduction from within the continent (cross-border transmission or within-country persistence). Persistence and trans-national transmission are a function of the number of cholera cases attributable to lineage *v*, calculated from prevalence of lineage *v* ($\lambda_{v,j,t-1}^{*}$) and total observed cases $c_{j,t-1}$, in country *j* and year *t −* 1, and connectivity between locations. Prevalence is calculated relative to other circulating lineages

$$\lambda_{v,j,t-1}^{*}=\frac{\lambda_{v,j,t-1}\alpha_{v,j,t-1}}{\sum_{v}\lambda_{v,j,t-1}\alpha_{v,j,t-1}}$$ (8)

where

$$\alpha_{v,j,t-1}=\text{Pr}\left(z_{v,j,t-1}=1|z_{v,j,t-2}=k\right)$$ (9)

Connectivity between locations, $\xi_{j,i}$, is modeled using a gravity model for locations j≠i, otherwise is the self-persistence parameter, δ

$$\text{log}\left(\xi_{j,i}\right)=\left\{\begin{array}{cc} \text{log}\left(\delta \right) & \text{if}\;j=i\\ \text{log}\left(\kappa \frac{\text{po}p_{j}^{\tau_{d}}\text{po}p_{i}^{\tau_{r}}}{d_{j,i}^{\zeta }}\right)+\omega_{j,i} & \text{if}\;j\neq i \end{array}\right.$$ (10)

where κ is the gravity constant, pop*_i_* is the population size in country *i*, $d_{j,i}$ is the Euclidean distance between centroids of countries *i* and *j* in kilometers, $\omega_{j,i}$ represents the random effect, $\tau_{d}$ and $\tau_{r}$ are scaling factors for population size for donor and recipient countries, respectively, and ζ is a scaling factor for distance between country centroids.

We fit the spatial random effect using a mixture model designed to moderately downweight gravity-based connectivity between locations in the absence of genomic and epidemiologic evidence of connection, where $\theta_{k}=0.7,\mu_{k}=\left[-3,0\right],\sigma_{k}=\left[1,1\right]$, based on functional form analysis

$$\text{Pr}\left(\omega_{j,i}|\theta_{k},\mu_{k},\sigma_{k}\right)=\sum_{k}\theta_{k}\text{Normal}\left(\mu_{k},\sigma_{k}\right)$$ (11)

Last, intercontinental introduction, $\gamma_{v,i,t}$, was calculated on the basis of inferred introduction timings ($\gamma_{v,t}$) and locations ($\gamma_{v,i}$) of lineages

$$\gamma_{v,i,t}=\gamma_{v,t}\times \gamma_{v,i}$$ (12)

#### Forward, backward, and forward-backward algorithm

With the elements of the HMM as defined above, we then implemented the forward algorithm to calculate filtered probabilities of presence, $\text{Pr}(z_{v,i,t}=1|y_{1:t})$ (*37*)

$$\text{Pr}(z_{v,i,t}=1|y_{1:t-1})=\sum_{k\in \left\{0,1\right\}}\Phi_{v,i,t}^{k,l}\text{Pr}(z_{v,i,t}=1|y_{1:t-1})$$ (13)

$$\psi_{v,i,t}=\text{Pr}\left(y_{v,i,t}|z_{v,i,t}\right)$$ (14)

$$\alpha_{v,i,t}^{l}=\alpha_{v,i,t-1}^{k}\Phi_{v,i,t}^{k,l}\psi_{v,i,t}$$ (15)

$$\alpha_{v,i,t}=\text{Pr}\left(z_{v,i,t}=1|z_{v,i,t-1}=k\right)$$ (16)

where $\alpha_{v,i,t}$ is the forward probability of presence, $\Phi_{v,i,t}^{k,l}$ is the transition probability (i.e., probability of introduction/reintroduction into country i at time t), and $\psi_{v,i,t}$ is the observation process.

The backward algorithm was implemented as

$$\beta_{v,i,t}\left(l\right)=\text{Pr}\left(y_{v,i,t+1:T}|z_{v,i,t}=l\right)$$ (17)

$$\begin{array}{c} \beta_{v,i,t-1}\left(k\right)=\text{Pr}\left(y_{v,i,t:T}|z_{v,i,t}=k\right)\\ =\sum_{l=1}^{L}\text{Pr}\left(y_{v,i,t+1:T}|z_{v,i,t}=l\right)\text{Pr}\left(y_{t}|z_{v,i,t}=l\right)\text{Pr}\left(z_{v,i,t}=l|z_{v,i,t-1}=k\right)\\ =\beta_{v,i,t}\psi_{v,i,t}\Phi_{v,i,t}^{k,l} \end{array}$$ (18)

With the backward algorithm specified, we define the forward-backward algorithm for the smoothed probability of presence/absence of a specific lineage in a given country and year $\left(\rho_{v,i,t}^{k}\right)$ as

$$\begin{array}{cc} \rho_{v,i,t}^{k} & =\text{Pr}\left(z_{v,i,t}=l|y_{1:T}\right)\\ & =\frac{\alpha_{v,i,t}\left(l\right)\beta_{v,i,t}\left(l\right)}{\text{Pr}\left(y_{1:T}\right)}\\ & \approx \alpha_{v,i,t}\left(l\right)\beta_{v,i,t}\left(l\right) \end{array}$$ (19)

#### Parameter priors

Initial probability

$$\pi_{v,i,1}∼\left\{\begin{array}{l} \text{Beta}\left(a_{\pi_{1}},b_{\pi_{1}}\right):E\left(\pi_{1}\right)=0.98,\sigma_{\pi_{1}}=0.02,\;y_{v,i,t}>0\\ \text{Beta}\left(a_{\pi_{2}},b_{\pi_{2}}\right):E\left(\pi_{2}\right)=0.43,\sigma_{\pi_{2}}=0.18,\;y_{v,t}>0\\ \text{Beta}\left(a_{\pi_{3}},b_{\pi_{3}}\right):E\left(\pi_{3}\right)=0.17,\sigma_{\pi_{3}}=0.10,\;y_{v,t-}>0\\ \text{Beta}\left(a_{\pi_{4}},b_{\pi_{4}}\right):E\left(\pi_{4}\right)=0.02,\sigma_{\pi_{4}}=0.02,\;y_{v,t-}=0 \end{array}\right.$$

*Observation process.* Case underreporting by country was fit on the basis of the following prior distribution

$$\varepsilon_{i}∼\text{Beta}(a_{\varepsilon },b_{\varepsilon )}:E\left(\varepsilon \right)=0.8,\sigma_{\varepsilon }=0.1$$

*Transition process.* The connectivity parameters were fit on the basis of the following prior distributions

$$\eta ∼\text{Beta}\left(a_{\eta },b_{\eta }\right):E\left(\eta \right)=0.45,\sigma_{\eta }=0.002$$

κ∼Normal(0.5,0.1)

log(δ)∼Normal(−3,0.5)

$$\tau_{d}∼\text{Beta}\left(a_{\tau_{d}},b_{\tau_{d}}\right):E\left(\tau_{d}\right)=0.45,\sigma_{\tau_{d}}=0.002$$

$$\tau_{r}∼\text{Beta}\left(a_{\tau_{r}},b_{\tau_{r}}\right):E\left(\tau_{r}\right)=0.35,\sigma_{\tau_{r}}=0.002$$

ζ∼Normal(2.25,0.2)

Priors were chosen on the basis of functional form analyses, with gravity priors informed by literature estimates of gravity parameters for epidemic spread (*38*), including cholera transmission (*39*). Tight priors were required for case and population transformation parameters for model convergence. Priors for lineage introduction timings ($\gamma_{v,t}$) and locations ($\gamma_{v,i}$) from outside of the continent were based on time to most recent common ancestor findings from Weill *et al.* (*12*) for lineages AFR1 to AFR8 and our phylogeographic analysis for lineages AFR9 to AFR17 (table S5).

#### Model diagnostics

The HMM model was run using four chains with 2000 warmup and 2000 sampling iterations. Chain convergence was assessed by whether any divergent transitions occurred, visual inspections of trace plots and posterior parameter distributions, R-hat values (≤1.1), and ESS.

#### Postestimation

We filled in the gaps in the observed phylogenetic lineage data by inferring the prevalence of each lineage across space and time and inferring the most likely sequence of (presence) states for each lineage using the Viterbi algorithm (*40*). First, using smoothed predicted probabilities of presence and predicted lineage prevalence, we inferred the likely number of cases attributed to each lineage across space and time based on a multinomial draw

$$c_{v,i,t}^{*}=\text{multinomial}\left(V,\lambda_{i,t}^{*}\right)$$ (20)

and determined lineage-specific prevalence as

$$\lambda_{v,i,t}^{\rho^{+}}=\lambda_{v,i,t}^{*}\rho_{i,t}^{+}$$ (21)

where

$$\rho_{i,t}^{+}=1-\prod_{v}1-\rho_{v,i,t}$$ (22)

We then used the Viterbi algorithm (*37*, *40*) to infer the most likely sequence of states for each lineage across space and time. This algorithm uses maximum a posteriori estimation to jointly infer the single most likely state sequence, where the most probable path is calculated moving forward in time, conditioning on the most likely state determined for the previous time step to calculate the most likely state at time *t*

$$z_{i,t}^{*}={\text{argmax}}_{z_{v,i,1:T}}\text{Pr}\left(z_{v,i,1:T}|y_{v,i,1:T}\right)$$ (23)

### Defining “cholera transmission units”

To define epidemiologically relevant cholera transmission units, we used transition rates from the full posterior of the phylogeographic analysis and inferred trans-national connectivity from the full posterior from the HMM model. For each analysis, we used the Louvain algorithm for community detection (*18*) to define nonoverlapping communities on the full network, using transition rates and inferred connectivity as edge weights for the phylogeographic and HMM analyses, respectively. This method of community detection produces a distribution of the proportion of times a country-pair is assigned to the same community (fig. S1). We then used a consensus clustering algorithm (*19*) to define cholera transmission units for both phylogeographic and HMM analyses. Briefly, to implement this algorithm, we use the proportion of times a country-pair is assigned to the same community as edge weights, with a threshold τ of 0.15 for edge weights. Any edge weight below the threshold is assigned a weight of 0, and the algorithm is run until there are only two possible edge weights (0 or 1), with each country now assigned to a consensus cluster. We confirmed the assignment of cholera transmission units using a divisive clustering algorithm where we defined the dissimilarity matrix based on the output of the proportion of times a country-pair clustered together from the Louvain algorithm, with the number of clusters (*k*) chosen on the basis of the silhouette method, finding nearly identical consensus clusters between the two methods.

### Simulation of cholera spread

Using inferences from our HMM model, we then conducted simulations to predict dynamics of cholera spread based on introductions or reintroductions of a cholera lineage into each country. We first simulated the 1-year risk of an outbreak in countries across the continent based on an introduction or reemergence in each country. This simulation was set up on the basis of the transition process from our HMM model, defined above. Briefly, we defined the probability of an outbreak $p_{i,t=1}$ in each country i in year t=1 based on the number of cases in the country with an outbreak (source country, j) in year t−1 and trans-national connectivity country $\xi_{i,j}$

$$p_{i,t}=\text{binomial}\left(1,\alpha_{i,t}\right)$$ (24)

$$\alpha_{i,t}=1-\Phi_{i,t}$$

$$\Phi_{i,t}=e^{-\phi_{i,t}}$$

where

$$1-e^{-\phi_{i,t}}=\text{Pr}\left(z_{i,t}=1|z_{j,t-1}=k\right)$$

$$\phi_{i,t}={\left(c_{j,t-1}\right)}^{\eta }\xi_{j,i}$$

The cases in the source country (j) were sampled from a Poisson distribution based on the observed distribution of reported suspected cholera cases in country j from 1990 onward, due to sparse reporting in early years.

We then examined the downstream impacts of an outbreak due to a new or reintroduction of a lineage in country i, simulating the speed of spread across the continent. This simulation was conducted similarly to the previous simulation, except that the full network of trans-national connectivity was taken into account and the simulation was run over 30 years

$$p_{i,t}=\text{binomial}\left(1,\alpha_{i,t}\right)$$ (25)

$$\alpha_{i,t}=1-\Phi_{i,t}$$

$$\Phi_{i,t}=\prod_{j}e^{-\phi_{i,t}}$$

where

$$1-e^{-\phi_{i,t}}=\text{Pr}\left(z_{i,t}=1|z_{j,t-1}=k\right)$$

$$\phi_{i,t}={\left(c_{j,t-1}\right)}^{\eta }\xi_{j,i}$$

where, again, cases were sampled from a Poisson distribution based on the distribution of cases observed in country j from 1990 onward.

The output from this simulation contained the distribution of the timing of arrival of a cholera lineage in each country i given an outbreak or introduction/reintroduction in country j. We ran 2000 simulations for each country of introduction (45 countries) for each scenario. Parameters $\xi_{j,i},\eta ,\;\text{and}\;\delta$ were sampled from the full posterior of the HMM model.

### Sensitivity analyses

First, cholera transmission dynamics likely evolve through time as risk factors change. Therefore, we subset the data to observations from 1990 onward (93% of sequences) and from 2000 onward (76% of sequences) and reran the HMM model and clustering analysis outlined above.

Next, to evaluate our model’s ability to recover missing lineage information, we created multiple partitions where we downsampled outbreaks with sequence availability by 20%, meaning that we removed information about detected lineages from 20% of reported outbreaks. We then reran the HMM model on each partition and determined the number of false negatives (known lineage presence but not recovered by the model), false positives assuming clonal outbreaks (a lineage different from the observed detected lineage was inferred by the model in addition to the originally detected lineage), and false positives assuming nonclonal outbreaks [allowing inferred lineages in addition to lineage(s) detected through sequencing in observed data]. We then calculated mean recall and precision assuming (i) clonal outbreaks and (ii) nonclonal outbreaks across partitions (table S2).

Last, to evaluate our predictions from speed of spread simulations, we compared simulated arrival times to observed arrival times. We determined observed arrival times as the time between estimated median (and bounds of 95% HPD) year of arrival into a likely country of introduction, based on findings from Weill *et al.* (*12*) for early lineages, and our phylogeographic analysis for later lineages, and (i) the first year a lineage was detected in countries where the lineage was observed or (ii) the first year a lineage was inferred to be present based on our HMM model. We calculated agreement between observed/inferred arrival times and median simulated arrival times using the Pearson correlation coefficient.

### Software

Data cleaning, postestimation analyses, and figure generation were conducted in R v4.5.2 (*41*). We used Stan (*42*) to run the HMM models, using cmdstanr v0.9.0 (*43*) to interface with cmdstan v2.38.0. Code used in analyses is available on GitHub (https://github.com/UNCIDD/cholera-units) and Zenodo (https://doi.org/10.5281/zenodo.20736318).
