# Supplementary material for: Defining epidemiologically relevant units of cholera transmission in sub-Saharan Africa
Source: Sci Adv. 2026 Nov 4;12(45):eaeb4683. doi: 10.1126/sciadv.aeb4683 (PMC13733893; doi:10.1126/sciadv.aeb4683)
Supplement: Supplementary file 1 [file sciadv.aeb4683_sm.pdf]

Science Advances
AAAS

Supplementary Materials for

Defining epidemiologically relevant units of cholera transmission in sub-Saharan Africa

Bethany L. DiPrete *et al.*

Corresponding author: Bethany L. DiPrete, diprete@email.unc.edu

Sci. Adv. **12**, eaeb4683 (2026)
DOI: 10.1126/sciadv.aeb4683

The PDF file includes:

Figs. S1 to S7
Legend for table S1
Tables S2 to S5

Other Supplementary Material for this manuscript includes the following:

Table S1

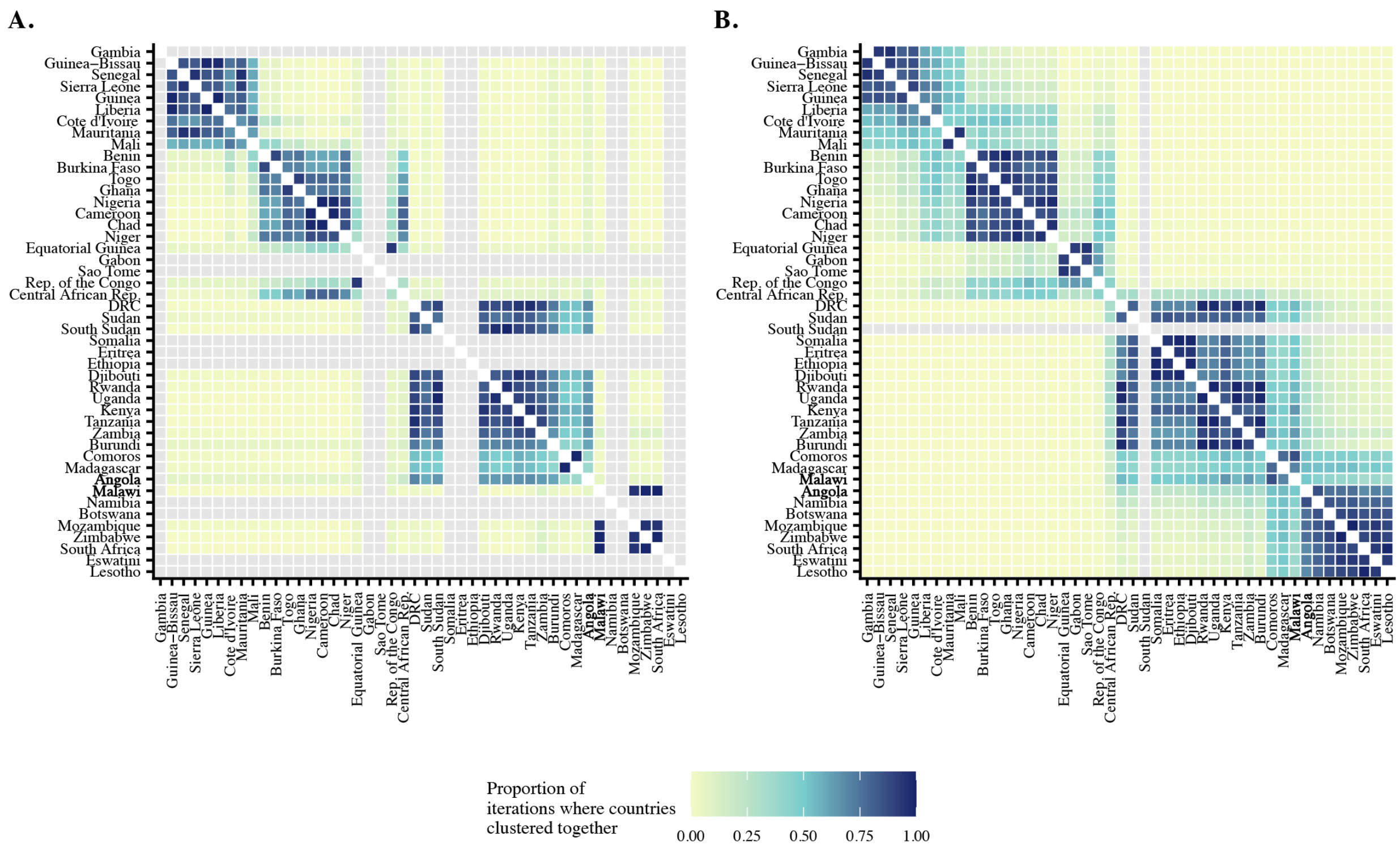

Fig. S1. Proportion of iterations where country-pairs clustered together. Heatmaps of the proportion of runs where country-pairs were grouped together in nonoverlapping neighborhoods using the Louvain method to define neighborhoods. (A) phylogeographic analysis, where an iteration refers to a state in the posterior. Bolded countries are in a different order than panel B due to clustering. (B) HMM analysis. Bolded countries are in a different order than panel A due to clustering.

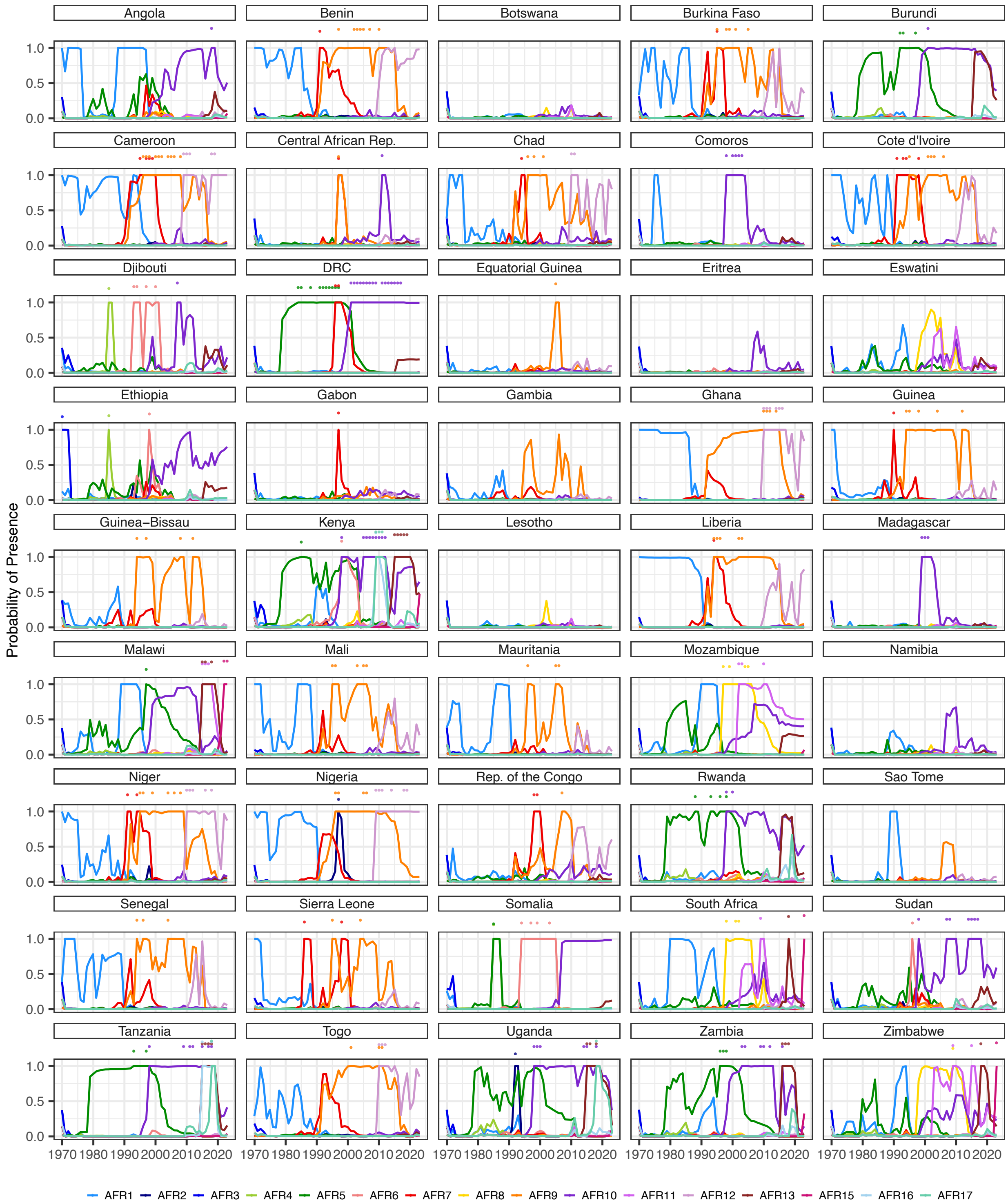

Fig. S2. Inferred probability of presence of cholera lineages over time by country. Facet panels show the mean probability of presence of each cholera lineage by year and country from the full posterior, with points in the top of each panel indicating whether a lineage was detected in that country and year.

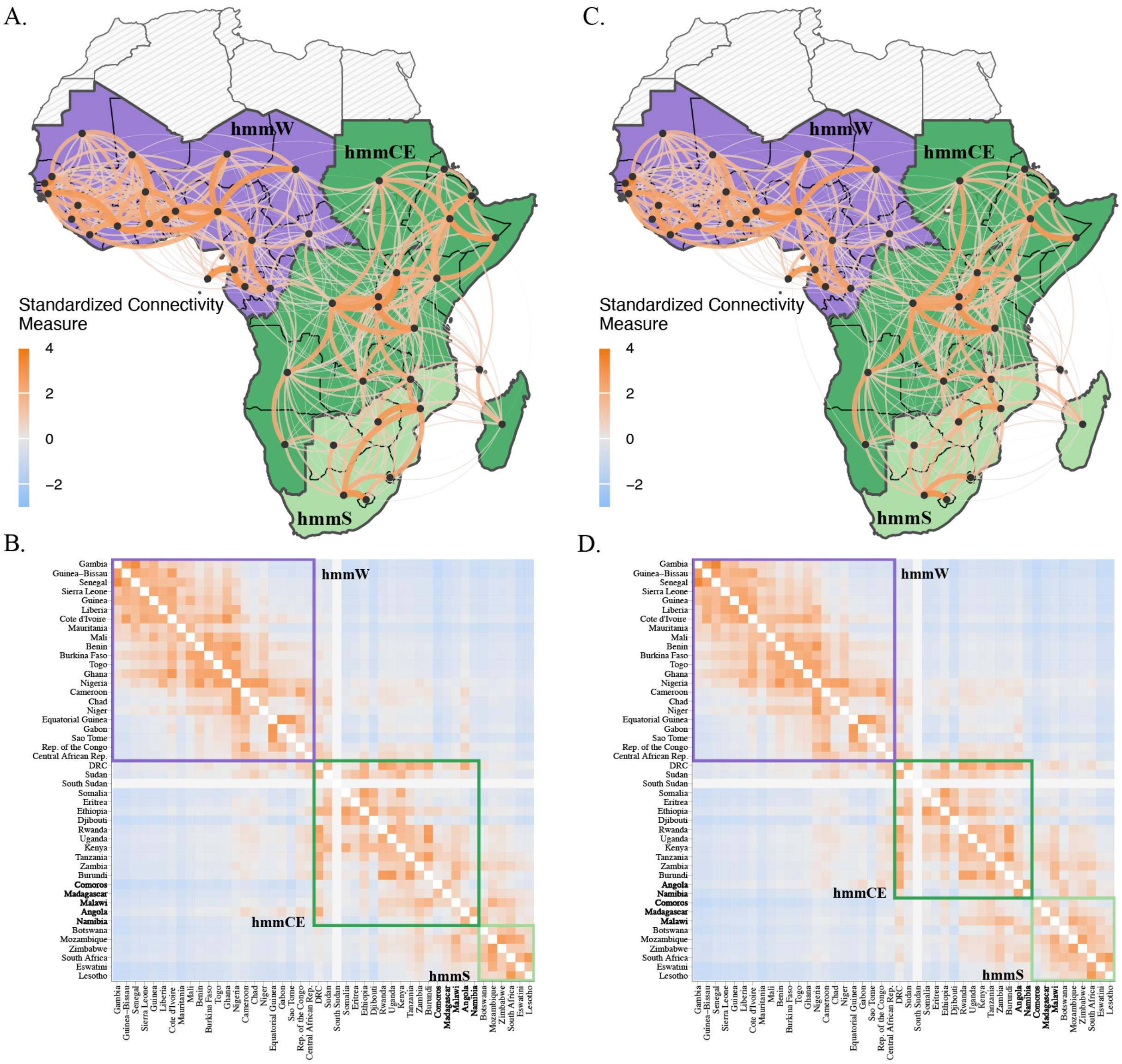

Fig S3. Inferred cholera transmission units and cross-country transmission in sub-Saharan Africa. (A) Results from HMM analysis where data was subsetted to 1990 onwards. Three identified cholera transmission units are shown by fill color (purple - hmmW, dark green - hmmCE, light green - hmmS), with the color and thickness of edges representing the standardized connectivity measure between countries. Only the top 50% of edges are plotted. **(B)** Heat map of the standardized connectivity measure between countries using the full network. Country names that are bolded are in a different order than panel D based on clustering. **(C)** Results from HMM analysis where data was subsetted to 2000 onwards. Three identified cholera transmission units are shown by fill color (purple - hmmW, dark green - hmmCE, light green - hmmS), with the color and thickness of edges representing the standardized connectivity measure between countries. Only the top 50% of edges are plotted. **(D)** Heat map of the standardized connectivity measure between countries using the full network. Country names that are bolded are in a different order than panel B based on clustering.

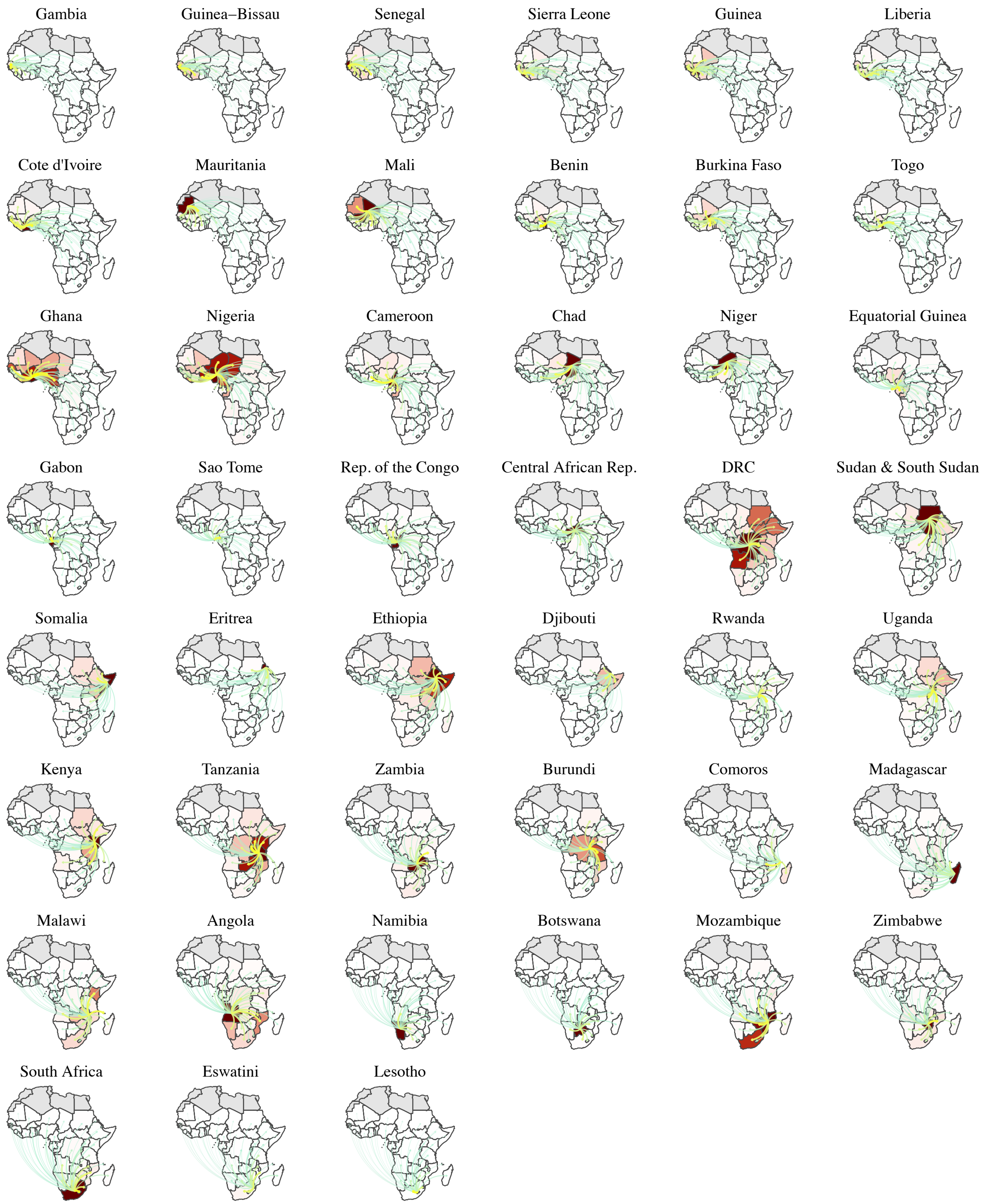

Fig. S4. One-year risk of cholera outbreak by country of introduction. Simulated one-year risk of cholera outbreaks from 2,000 introductions of cholera into each of 45 countries (sampling parameters from the posterior for each), with darker red indicating higher risk of an outbreak in the following year. Edges indicate the asymmetrical strength of connectivity between the country of introduction and the rest of the continent.

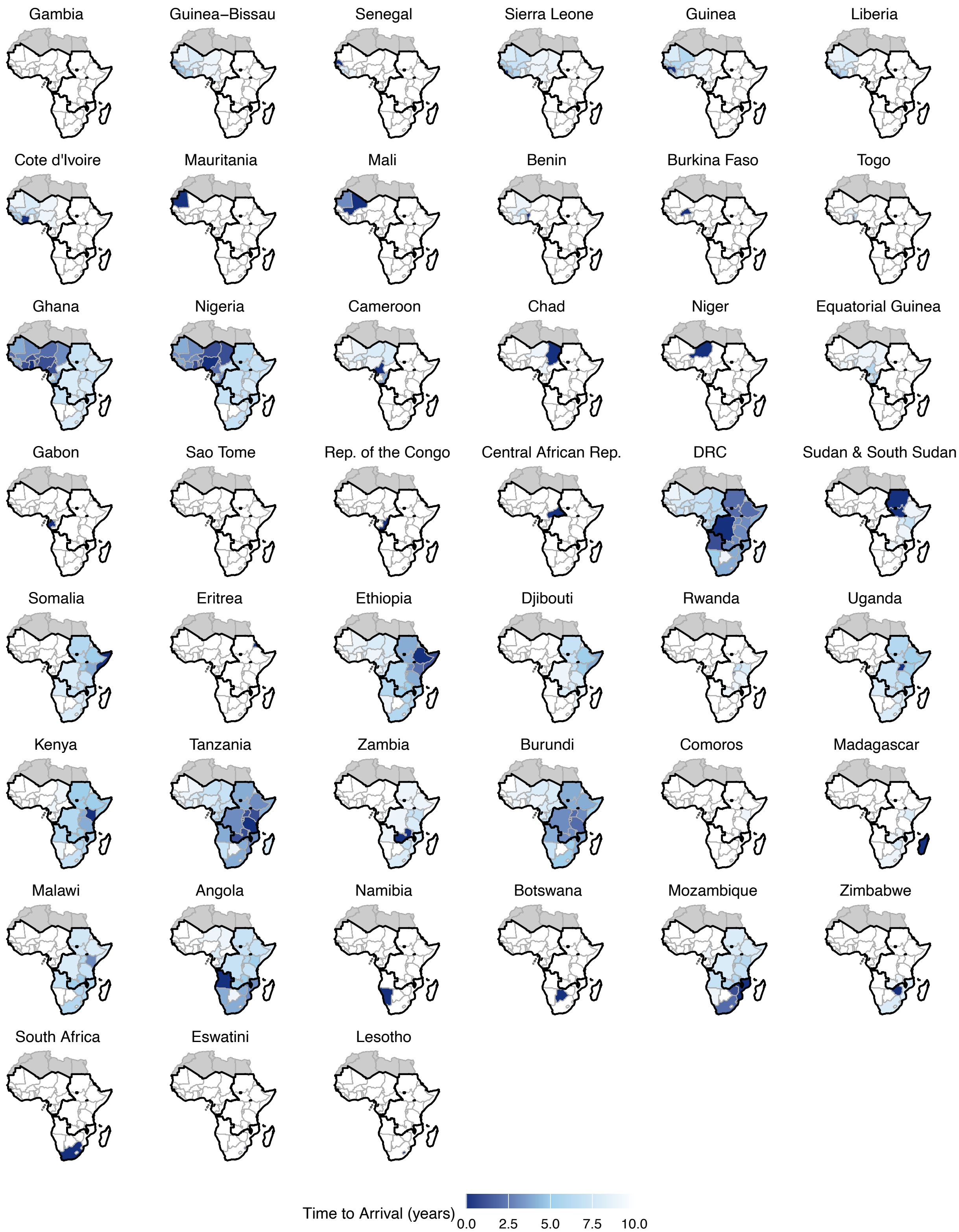

Fig. S5. Speed of cholera spread by country of introduction. Simulated downstream time to arrival of cholera from 2,000 introductions of cholera into each of 45 countries (sampling parameters from the posterior for each).

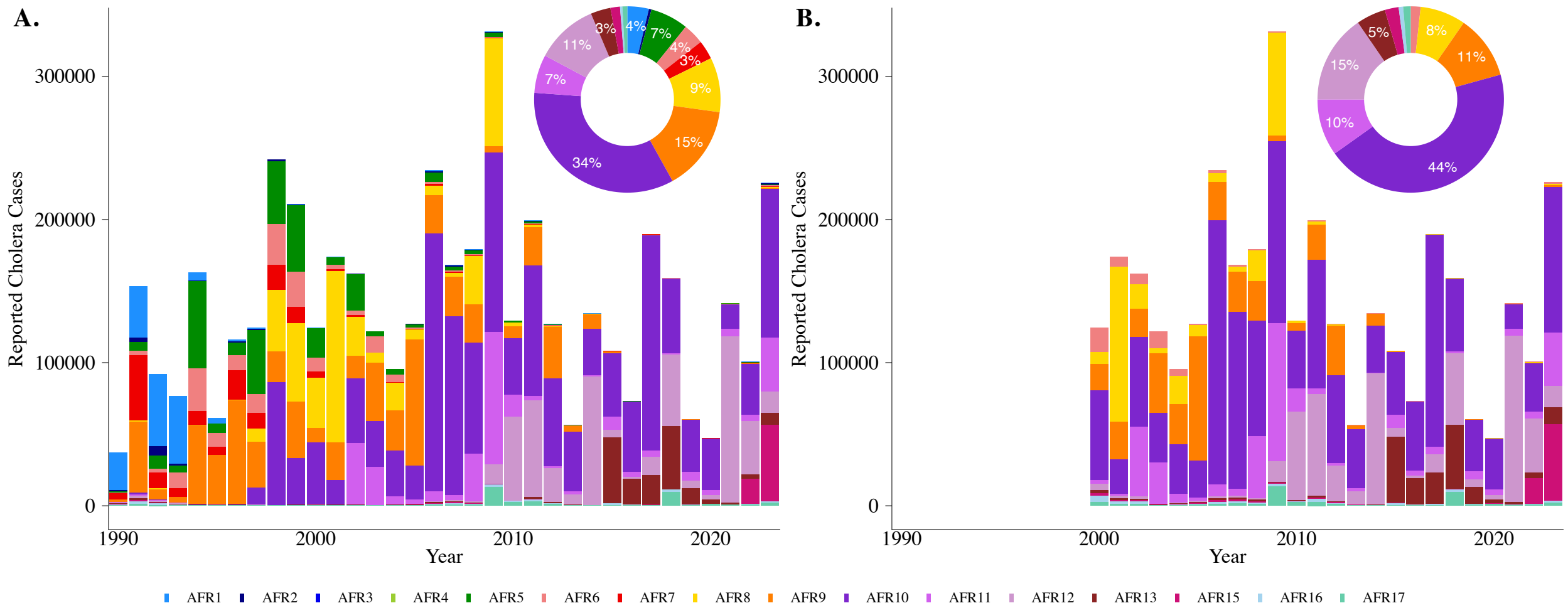

Fig. S6. Lineage prevalence. Bar graph indicates total cases by year attributable to a given observed lineage or lineage inferred from the HMM model, and donut plot shows the proportion of cases attributable to each lineage overall in **(A)** data subset to 1990 onwards, and **(B)** data subset to 2000 onwards.

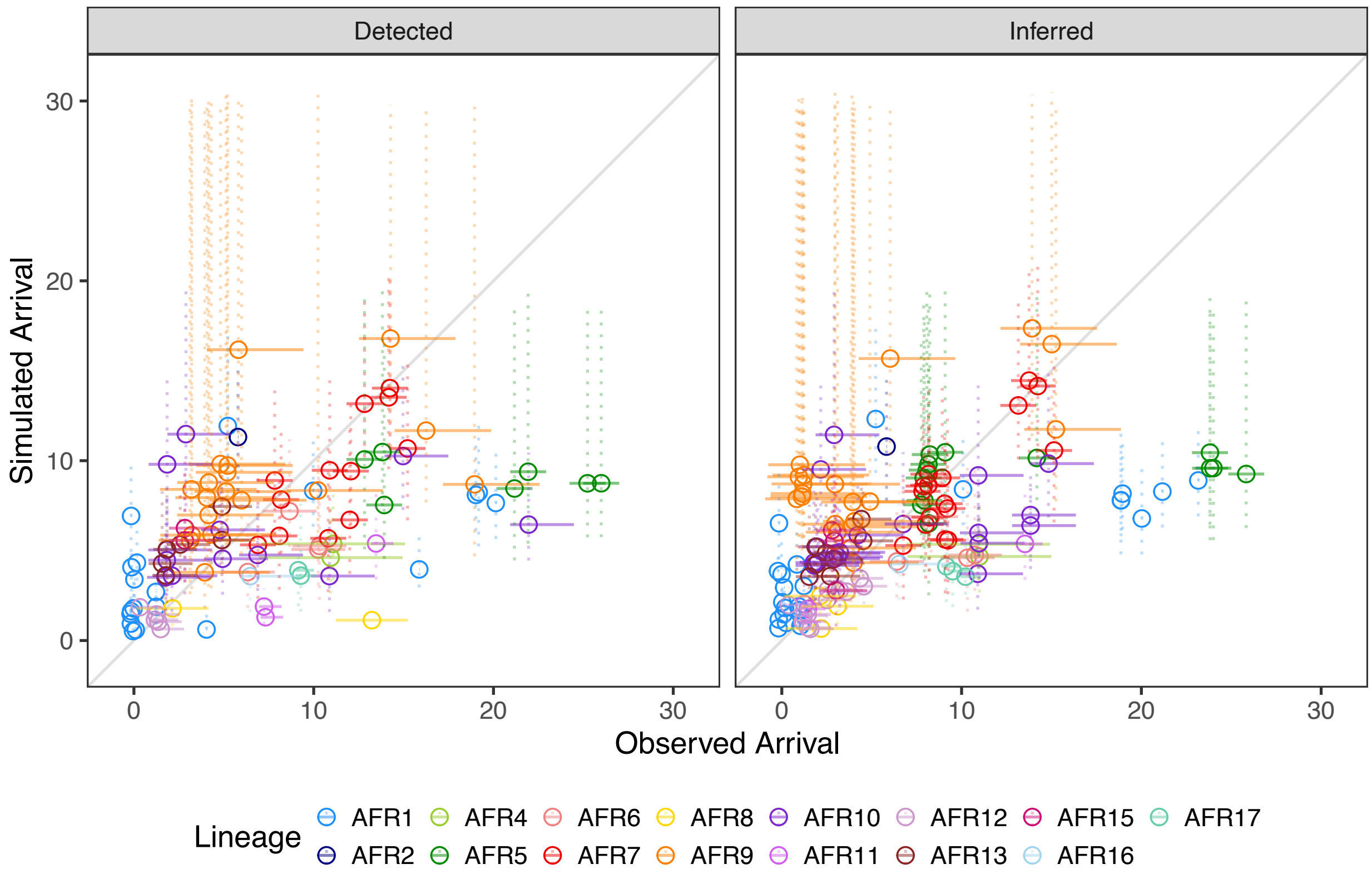

Fig. S7. Observed vs simulated time to arrival by lineage. Comparing median (IQR shown by dashed vertical line) simulated arrival times of lineages to **(left)** observed lineage arrival time, determined by inferred country and median (solid line depicts 95% HPD) year of introduction to the continent from phylogeographic analyses and first year the lineage was detected by country, and **(right)** inferred lineage arrival time, determined by inferred country and median (solid line depicts 95% HPD) year of introduction to the continent from phylogeographic analyses and first year the lineage was inferred by the HMM model to have arrived by country.

Table S1 (separate .csv file). List of accession numbers and metadata for sequences used in this analysis.

Country with an Outbreak	Expected Outbreaks Caused in Additional countries in 1 Year	Outbreaks Caused Within vs. Between Transmission Units		Outbreaks by Transmission Unit		
		Between Units	Within Unit	hmmW	hmmS	hmmCE
Angola	1.23	0.65	0.58	0.18	0.58	0.47
Benin	0.43	0.02	0.41	0.41	0.01	0.01
Botswana	0.01	0.00	0.01	0.00	0.01	0.00
Burkina Faso	0.41	0.01	0.40	0.40	0.00	0.01
Burundi	1.48	0.16	1.32	0.03	0.13	1.32
Cameroon	0.67	0.10	0.58	0.58	0.03	0.06
Central African Rep.	0.09	0.05	0.04	0.04	0.01	0.04
Chad	0.36	0.09	0.27	0.27	0.02	0.08
Comoros	0.42	0.05	0.37	0.01	0.04	0.37
Cote d'Ivoire	0.79	0.01	0.78	0.78	0.0	0.01
DRC	0.41	0.02	0.39	0.01	0.0	0.39
Djibouti	2.88	1.14	1.73	0.43	0.71	1.73
Equatorial Guinea	0.85	0.06	0.79	0.79	0.02	0.05
Eritrea	0.03	0.00	0.03	0.00	0.00	0.03
Eswatini	0.09	0.02	0.07	0.00	0.07	0.01
Ethiopia	2.46	0.23	2.23	0.16	0.07	2.23
Gabon	0.15	0.01	0.13	0.13	<0.01	0.01
Gambia	0.11	0.00	0.11	0.11	0.00	0.00
Ghana	4.75	0.06	4.69	4.69	0.02	0.04
Guinea	0.93	0.02	0.91	0.91	0.01	0.01
Guinea-Bissau	0.75	0.01	0.74	0.74	0.0	0.01
Kenya	1.08	0.15	0.93	0.07	0.08	0.93
Lesotho	0.00	0.00	0.00	0.00	0.00	0.00
Liberia	0.53	0.02	0.51	0.51	0.01	0.01
Madagascar	0.46	0.17	0.28	0.03	0.14	0.28
Malawi	1.02	0.38	0.64	0.05	0.33	0.64
Mali	0.54	0.02	0.52	0.52	0.00	0.02
Mauritania	0.17	0.01	0.16	0.16	<0.01	<0.01
Mozambique	2.04	0.40	1.64	0.06	1.64	0.34
Namibia	0.09	0.03	0.06	0.01	0.06	0.02
Niger	0.28	0.04	0.24	0.24	0.01	0.03
Nigeria	4.53	0.19	4.33	4.33	0.06	0.14
Rep. of the Congo	0.25	0.07	0.18	0.18	0.02	0.05
Rwanda	0.28	0.04	0.24	0.02	0.02	0.24
Sao Tome	0.08	0.01	0.07	0.07	0.00	0.01
Senegal	1.00	0.02	0.98	0.98	0.01	0.01
Sierra Leone	0.73	0.02	0.71	0.71	0.01	0.02
Somalia	0.63	0.09	0.54	0.06	0.03	0.54
South Africa	0.47	0.13	0.34	0.04	0.34	0.09
Sudan & South Sudan	0.65	0.23	0.42	0.20	0.03	0.42
Tanzania	3.96	0.40	3.55	0.10	0.30	3.55
Togo	0.27	0.01	0.26	0.26	0.0	0.01
Uganda	0.69	0.13	0.56	0.08	0.05	0.56
Zambia	0.48	0.22	0.26	0.05	0.17	0.26
Zimbabwe	0.43	0.21	0.22	0.05	0.22	0.16

Table S2. Expected number of outbreaks caused in additional countries within one year by country of introduction.

	**Predicted Positive** (Present)	**Predicted Negative** (Absent)
**Actual Positive** (Present, assuming clonal)	240	88
**Actual Negative** (Not present, assuming clonal)	130	-
	**Recall** = 73% (SD = 7)	
	**Precision** = 65% (SD = 7)	
	**Predicted Positive** (Present)	**Predicted Negative** (Absent)
**Actual Positive** (Present, assuming clonal)	240	88
**Actual Negative** (Not present, assuming nonclonal)	42	-
	**Recall** = 73% (SD = 7)	
	**Precision** = 85% (SD = 8)	

Table S3. **Validation across downsampled partitions, each with 20% downsampling**. Table shows recovery (inferred presence or absence) of detected lineages, recall, and precision assuming clonal outbreaks (top) or nonclonal outbreaks (bottom).

Parameter	Interpretation
$z_{v,i,t}$	Hidden state of lineage presence or absence
$y_{v,i,t}$	Observed lineages from genomic sequencing
$\pi_{v,i,1}$	Initial probability of presence
$\mathbf{\Phi}$	Transition process
$\mathbf{\Psi}$	Observation process
$\psi_{v,i,t}$	Probability of observed data given hidden state
$\rho_{v,i,t}$	Probability of lineage presence
$\lambda_{v,i,t}$	Sequences attributable to lineage v
$\lambda^*_{v,i,t}$	Prevalence of lineage v
$\alpha_{v,i,t}$	Forward probability of presence
$\mathbf{A}$	Transition matrix
$\Phi^{k,l}_{v,i,t}$	Probability of transition from state k to state l
$\phi_{v,j,t}$	Transmission rate from country j to country i
$c_{i,t}$	Cases in country i in year t
$\xi_{i,j}$	Connectivity between country i and country j
η	Scaling factor for cases
κ	Gravity constant
τ_d, τ_r	Scaling factors for donor and recipient countries, respectively
$d_{j,i}$	Euclidean distance (km) between centroids of country j and country i
ζ	Scaling factor for distance
$\omega_{j,i}$	Spatial random effect
δ	Within-country persistence
$\gamma_{v,i,t}$	Probability of introduction from outside of the continent
$\beta_{v,i,t-1}$	Backward probability of presence
$c^*_{v,i,t}$	Inferred cases attributable to each lineage

Table S4. Parameters used in the HMM model and their interpretations.

Lineage	***Timing*** *Median (95% HPD)*	***Location***
AFR9	1990.9393 (1989.5813, 1992.1127)	Guinea (94.7%), Nigeria (0.1%), Cote d'Ivoire (0.2%), Other (4.9%)
AFR10	1996.0134 (1995.1745, 1996.7141)	Kenya (90.7%), Tanzania (8.0%), DRC (0.4%), Uganda (0.5%), Other (0.5%)
AFR11	2001.7143 (2001.3565, 2001.9460)	Mozambique (98.9%), Zimbabwe (0.7%), Other (0.4%)
AFR12	2008.5967 (2008.2021, 2009.9100)	Nigeria (85.7%), Cameroon (14.1%), Niger (0.2%)
AFR13	2013.3436 (2012.6652, 2013.9457)	Kenya (94.4%), Tanzania (4.9%), Other (0.6%), Uganda (0.1%)
AFR15	2020.1102 (2019.5197, 2020.6738)	Malawi (95.8%), South Africa (4.0%), Other (0.2%)
AFR16	2008.5602 (2007.8748, 2008.9427)	Kenya (100.0%)
AFR17	2008.7018 (2008.3911, 2008.9303)	Kenya (99.3%), Other (0.7%)

Table S5. TMRCA estimates of introduction timing (median and 95% HPD) and location of *V. cholerae* lineages AFR9-AFR17.
